# The Use of Green Leaf Membranes to Promote Appetite Control, Suppress Hedonic Hunger and Loose Body Weight

**DOI:** 10.1007/s11130-015-0491-8

**Published:** 2015-06-27

**Authors:** Charlotte Erlanson-Albertsson, Per-Åke Albertsson

**Affiliations:** Department of Experimental Medical Science, Appetite Control Unit, Bio-Medical Centre (BMC), B11, Lund University, Sölvegatan 19, SE 221 84 Lund, Sweden; Department of Biochemistry and Structural Biology, Chemical Centre, Lund University, SE 221 00 Lund, Sweden

**Keywords:** Natural supplement, Thylakoids, Fat digestion, Satiety hormones, Gut microbiota

## Abstract

On-going research aims at answering the question, which satiety signal is the most potent or which combination of satiety signals is the most potent to stop eating. There is also an aim at finding certain food items or food additives that could be used to specifically reduce food intake therapeutically. Therapeutic attempts to normalize body weight and glycaemia with single agents alone have generally been disappointing. The success of bariatric surgery illustrates the rationale of using several hormones to treat obesity and type-2-diabetes. We have found that certain components from green leaves, the thylakoids, when given orally have a similar rationale in inducing the release of several gut hormones at the same time. In this way satiety is promoted and hunger suppressed, leading to loss of body weight and body fat. The mechanism is a reduced rate of intestinal lipid hydrolysis, allowing the lipolytic products to reach the distal intestine and release satiety hormones. The thylakoids also regulate glucose uptake in the intestine and influences microbiota composition in the intestine in a prebiotic direction. Using thylakoids is a novel strategy for treatment and prevention of obesity.

## Introduction

Obesity epidemic spreads rapidly over the world. The origin of obesity epidemic is clearly dependent on several parameters. One such is the increased consumption of energy-dense and nutrient-poor food, containing high levels of fat and sucrose [1].

Appetite control occurs through two systems, the homeostatic regulation and the hedonic regulation [2]. The homeostatic regulation involves control of energy intake and the hedonic regulation the control of sensory pleasure in eating. The loss of effective appetite control could either be due to a disturbance in the homeostatic pathway and/or an inappropriate sensitization of the hedonic pathway.

There is clearly an interaction between the homeostatic and hedonic systems [3]. Together they will modulate hunger and satiety but also the choice and liking of certain food items. To achieve appetite control and to be able to influence body weight regulation it is hence important to consider the processes responsible for energy homeostasis and the processes involved in the hedonics of eating [4]. One important factor contributing to appetite control is the bacterial flora in the intestine.

We have found that *thylakoids*, the photosynthetic membranes of green leaves, suppress hunger and promote satiety. This occurs through the modulation of gastrointestinal appetite peptides and a modulation of the gut microflora. In long-term studies the thylakoids produce weight loss together with a reduction of blood lipids and blood glucose. Subjects receiving thylakoids also have a decreased liking for fat and sweet. Thylakoids when added to food may therefore be helpful in achieving weight loss and improved health.

## Thylakoids - Properties and Composition

**Fig. 1:**
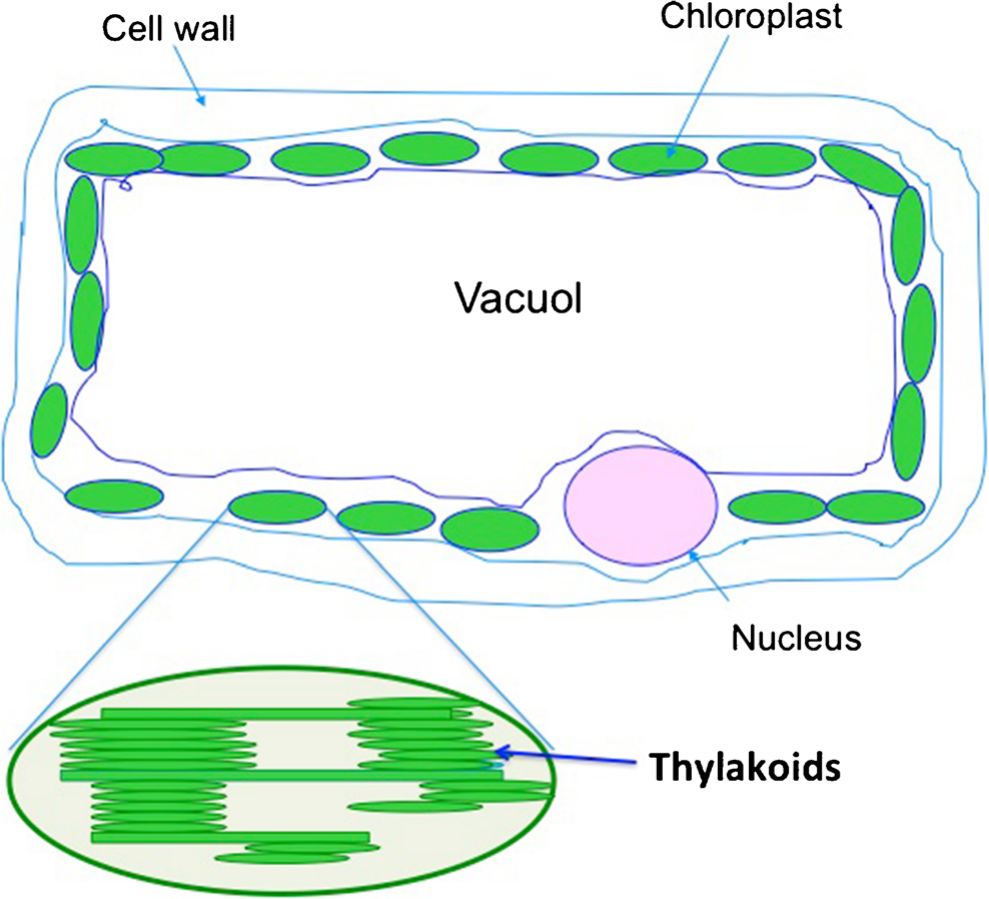
A plant cell consists of chloroplasts, where the photosynthesis takes place. The cell is surrounded by a cell wall, resistant to digestion by humans. A vacuole with water takes up 80 % of the volume of a cell. The thylakoids are membranes building up the photosynthetic apparatus in the chloroplast. The thylakoids consists of galactolipids, proteins, pigments, vitamins and antioxidants

Thylakoids are the membranes in the chloroplasts of green leaves responsible for the light reaction in photosynthesis (Fig. 1) [5, 6]. They are probably the most complex of biological membranes and organized into continuous paired membranes having different composition and function. The thylakoids contain over 100 different proteins, both intrinsic and extrinsic, together with membrane lipids and pigments. The thylakoids are located in chloroplasts of green leaves and at a higher concentration in dark green leaves compared to light green leaves. Characteristic of the proteins is that they are membrane spanning, which means that they are hydrophobic and attract to hydrophobic surfaces like lipids. The thylakoids have an iso-electric pH at 4,7 [7]. This means that they are positively charged at a pH below 4,7 and negatively charged above pH 4,7. In the stomach the pH is close to 2,0 during fasting conditions. During a meal the pH of gastric contents increases up to 6–7 depending on the food components and remains much above pH 2 until most of the meal is emptied. In the intestine pH is like-wise around 6,5 in the lumen. This means that the thylakoids are negatively charged and can bind positively charged ions. In the lumen of the intestine thylakoids form large swollen structures that adhere to the mucosal surface [8]. The mucosal surface of the intestine pH has an acidic pH, around 5,3 [9]. The thylakoids thus are rather isoelectric at the mucosal surface and hence easily adsorb to this, as has been demonstrated [8].

The thylakoids besides proteins contain phospholipids, galactolipids and pigments building up the membrane. The thylakoids are built as stacks, which connect to each other through a grana structure (Fig. 1). Together they form a tightly compressed and stable three-dimensional structure that is able to withstand outer forces from the environment but still retain a great flexibility. In the stomach and the intestine thylakoids are resistant to degradation by gastric and pancreatic enzymes [10]. This is an important property, since the thylakoids thereby remain in the intestine for several hours, before they get totally degraded. Through this stability food digestion in the presence of thylakoids goes on for a long time allowing the digestive products to reach the distal intestine to release satiety hormones. Pancreatic lipase-related protein 2, a lipase not being dependent on colipase, hydrolyses galactolipids present in vegetables [11, 12] and is responsible for the degradation of the thylakoids gradually occurring in the intestine. Thylakoids additionally contain vitamins E and K as well as certain pigments and antioxidants, like chlorophyll, carotenoids, zeaxantin and lutein. The antioxidants protect the plant against oxidation, induced by light, and may after consumption protect humans against disease. The bioavailablity of lutein and zeaxantin is high [13], meaning that the antioxidants may protect against disease, as evidenced by epidemiological studies. Chlorophyll is a green pigment that absorbs energy from light during the photosynthetic process present in green plants and algae. It has a structure similar to haemoglobin with a carbon chain, called phytol. There are many health claims about chlorophyll that has no substantial evidence. In scientific literature it has nevertheless been demonstrated to act as a protective agent against certain chemicals that induce cancer [14]. Moreover, chlorophyll or its metabolites may activate nuclear receptors, named PPARs [15]. These are nuclear receptors that activate genes regulating lipid metabolism, insulin sensitivity, and glucose homeostasis. When activated they stimulate fatty acid oxidation and decrease hyperlipidaemia [15]. High concentrations of chlorophyll in the intestine may therefore through activation of this nuclear receptor family activate fatty acid oxidation in the intestinal cell. The intestinal cell has the ability to oxidize fatty acids at a high rate [16]. Maximally 5 % of chlorophyll is absorbed in the intestine. Most chlorophyll passes through the colon and may affect gut health [17].

## Effects of Thylakoids

### Effect on Lipase/Colipase Activity

**Fig. 2:**
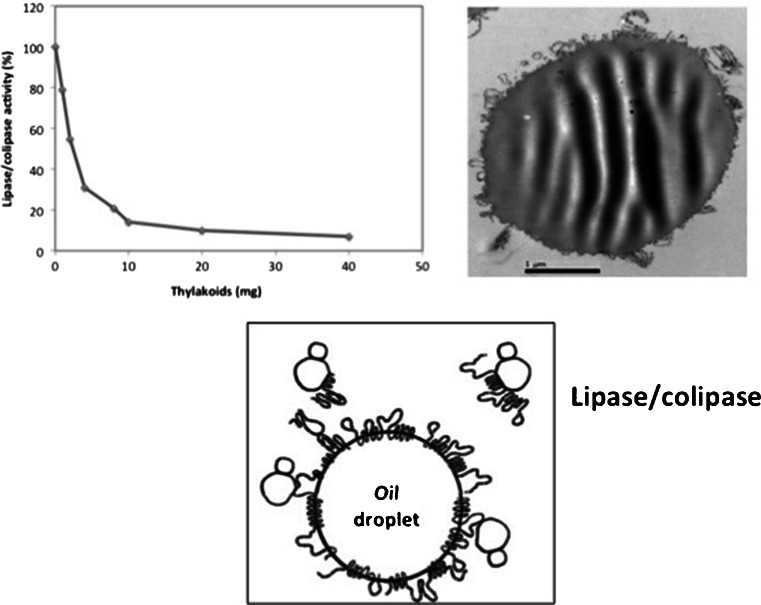
Thylakoids and fat digestion. Thylakoids inhibit lipase/colipase activity in a dose-dependent way [18]. The inhibition is due to the binding of thylakoids to the triglyceride interface, thus covering the substrate to be hydrolysed [18, 19]. The thylakoids also bind the pancreatic lipase/colipase complex [20]. The hydrolysis of the oil droplet thus occurs more slowly. Intestinal enzymes gradually break down the thylakoids, which allows fat digestion to be completed. Therefore there is no steatorrea

Our first discovery on the effect of thylakoids was a powerful inhibition of the pancreatic lipase/colipase catalysed hydrolysis of fat (Fig. 2) [18]. Pancreatic lipase with its protein cofactor colipase is the main enzyme responsible for hydrolysis of dietary fat in the intestine [21]. Lack of either lipase or colipase causes impaired fat digestion and steatorrea [22]. The idea that a reduced rate of fat digestion would affect appetite was clear from colipase knock out mice, who in their heterozygous form had a more slowly growth [23]. This idea was further promoted by the discovery of a compound that inhibited pancreatic lipase/colipase and at the same time reduced food intake in rat after oral administration [24].

The finding of thylakoids as inhibitors for lipase/colipase was based on the idea that galactolipids are powerful inhibitors of lipase/colipase, and that thylakoids are a rich source of galactolipids. Pancreatic lipase and colipase have been demonstrated to adsorb to galactolipids in monolayers [25], slowing down lipolysis, which may be important to promote satiety [26]. With our present knowledge there are several components in the thylakoids that are important for the observed appetite suppression, not only the galactolipids. These components are the proteins, *i.e*., light harvesting complexes and the antioxidants. The whole complex macromolecular structure of thylakoids is needed for an optimal effect on appetite suppression. The thylakoids display a dose-dependent inhibition of pancreatic lipase/colipase activity *in vitro*. To our surprise extraction of the lipids from the thylakoids, rendering a lipid free thylakoid product still had the ability to inhibit pancreatic lipase/colipase. Thus rather the hydrophobic proteins in the thylakoids are responsible for the observed inhibition [18].

Many proteins inhibit pancreatic lipase, but in the presence of bile salt and colipase, as occur during intestinal fat digestion under physiological condition, there is no such inhibition [27]. The ability of thylakoids to inhibit lipase even in the presence of bile salt and colipase is thus a completely new phenomenon. The inhibition is not unique to thylakoid membranes. We demonstrated this to be true also for other membranes, like plasma membranes from animal cells, mitochondria from animal cells and bacterial membranes [18]. Thylakoids are unique, because they are extremely resistant to proteolysis by gastric and pancreatic juice enzymes, in contrast to other membranes [10]. This resistance of thylakoids towards intestinal degradation is due to the high content of the membrane pigments chlorophyll a and b and carotenoids which are strongly bound to the intrinsic membrane proteins. The enzymes are thereby hindered to come in contact with the intrinsic membrane proteins. Such a stability of thylakoids is important for the effects on appetite, energy metabolism and intestinal bacterial flora. Mitochondria when added to food at a concentration to inhibit pancreatic lipase/colipase were unable to suppress food intake and body weight in long-term experiments in rat (unpublished experiments). Also the milk protein casein, which is a powerful inhibitor of pancreatic lipase/colipase failed to suppress food intake in rat. Thus, inhibition of pancreatic lipase/colipase *in vitro* subsequently does not necessarily mean a suppression of appetite and body weight *in vivo*.

The synthetic compound, diethylaminoethylether inhibited pancreatic lipase/colipase and at the same time suppressed appetite [24]. There was however no steatorrea, explained by an increased synthesis of pancreatic lipase leading to a persistent fat digestion at low rate [24]. There are several inhibitors for pancreatic lipase, with a potential use for obesity treatment. *Orlistat* (*Xenical*) reacts with the active site of lipase, in this way blocking lipase activity. The inhibition may be partially reversed in the presence of an emulsion [28]. When used clinically this compound has been demonstrated to cause body weight loss at an intermediate scale, around 5 kg per year. The side effects are steatorrea [29]. This is not a serious side effect as opposed to drugs that act centrally and has driven the use of *orlistat* into the field of reducing blood lipids and treating diabetes [30]. One basic difference between thylakoids and *orlistat* is the release of the satiety hormones, *e.g*., CCK by thylakoids [18, 31–33], but not with *orlistat* [34]. The reason is that the satiety hormones are released by fatty acids, which are the end products of triacylglycerol hydrolysis. Fatty acids are formed to 100 % from triacylglycerol with thylakoids, whereas with *orlistat* 25 % of the dietary fat leaves the intestine undigested. Undigested fat, i.e., triacylglycerol does not release any gastrointestinal satiety peptides. A complete fat digestion, although being at a reduced rate, is thus a prerequisite for optimal release of satiety hormones.

A large number of various polyphenols extracted from plants, fungi, algae, wine, green tea, and coffee have been found to inhibit pancreatic lipase when tested *in vitro* [35]. Several studies indicate anti-obesity effects in animal experiments, with a mechanism of action being an increased sympathetic activity, increased energy expenditure and increased oxidation of fat [36], as well as decreased nutrient absorption [37]. More studies are needed to document appetite and body weight suppression when administered to human.

Dietary fibres may promote satiety. Betaglucan from barley when added to white rice promotes satiety [38] and leads to suppressed food intake. Fibres can also bind dietary fat, which is then excreted in faeces. Another way of eliminating fat is by the addition of calcium ions. A high-calcium diet was found to excrete 10 g of fat daily compared to a low-calcium diet, leading to the excretion of 5 g of fat [39]. The mechanism is a binding of calcium to fatty acids, which are precipitated, and excreted through the faeces. Calcium also precipitates bile salt, which are needed for the uptake of fatty acids into the intestine.

There are various ways of increasing faecal fat excretion, neither of these mechanism hold for thylakoids. Thylakoids work by temporarily inhibiting pancreatic lipase/colipase during fat digestion. The delay in fat digestion causes the lipids to reach the distal end of the intestine, where they are absorbed. The overall important mechanism is that the food digestion is “forced” to reach the distal parts of the intestine. Thereby satiety hormones can be efficiently released. The mechanism for inhibition of pancreatic lipase/colipase is a binding of thylakoids to the lipid surface covering the triglyceride substrate to be hydrolysed (Fig. 2) [18]. Another mechanism is the binding of pancreatic lipase/colipase to thylakoids. Only a small percentage of pancreatic lipase/colipase is however bound to the thylakoids, suggesting that the inhibition of lipase/colipase activity is due to the covering of the triglyceride surface by thylakoids. Thylakoids have emulsifying properties, which means that they are attracted to lipid surfaces, forming stable emulsions of fat. In order to be active as appetite suppressor it is important that thylakoids are dissolved in a lipid formula, which make them optimally spread to form a monolayer around lipid particles and on surfaces.

### Promotion of Satiety and Suppression of Hunger

Administration of thylakoids inhibits eating and promotes satiety, as demonstrated in rat [18, 40], mouse [32] and human [31, 33, 41, 42]. Chronic administration of thylakoids effectively reduces body weight and improves glucose/lipid metabolism in overweight individuals, suggesting thylakoids to offer an effective therapeutic option for overweight patients. In animal studies thylakoids were mixed with food and in human studies thylakoids were mixed with food ingredients as part of a complete meal or served as a juice prior to a meal. Thylakoids are efficient in suppressing food intake irrespective of the composition of diet, *i.e.*, a high-fat [18, 31] or a high-carbohydrate diet [33, 40–42]. The suppression of food intake was a direct effect in human observed within the same meal [33, 42].

**Fig. 3:**
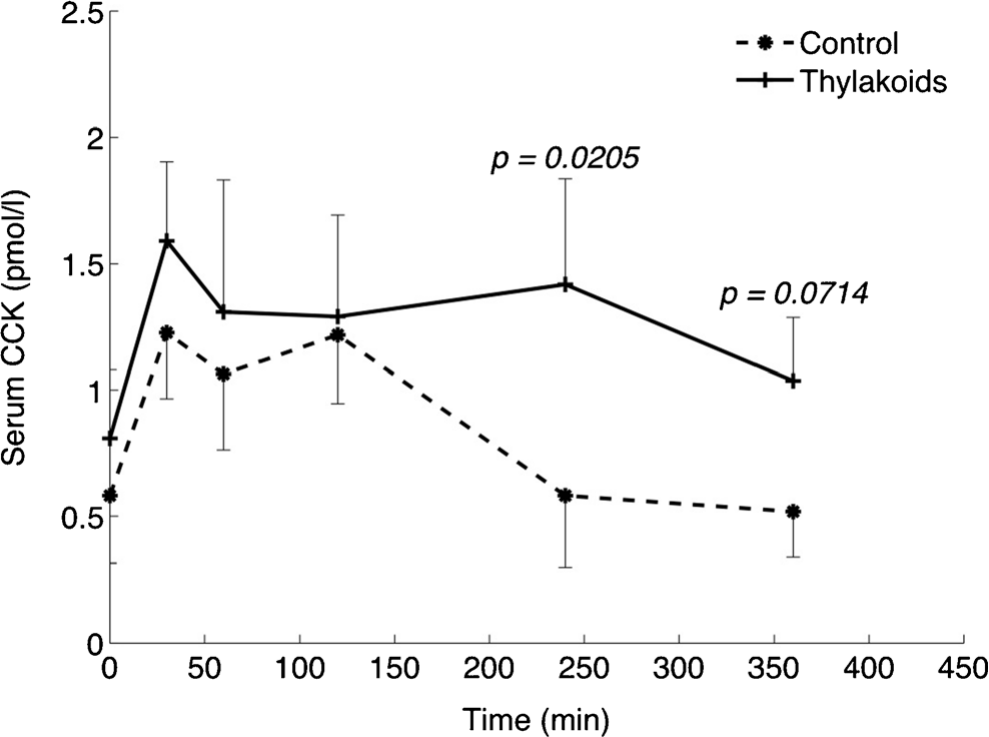
The release of CCK following a meal with and without thylakoids in human. With thylakoids there was a late release of CCK in addition to the early release. In control there was only an early release of CCK. Thus, thylakoids induce the release of the satiety hormone CCK during a longer period, being important for prevention of intermeal snacking [31]

In human a high-fat meal was served with increasing concentrations of thylakoids, from 5 to 50 g [31]. The thylakoids were mixed with rapeseed oil (26 g) and cashew-nuts (25 g), lime, salt and basil and was eaten as a pesto sauce on bread with tomatoes. The percentage fat was 65 energy %, carbohydrate 25 E% and protein10 E%. With such a meal CCK was released in a dose-dependent way, significantly different from control at time point 4 h and 6 h after the start of meal. The control meal rendered an optimal CCK release at time point 30 min after the start of meal, while with thylakoids there was an early release of CCK at time point 30 min and a new peak after 4 h (Fig. 3).

The time course suggests that the stimulus for release of CCK is continued up to 6 h. Since the meal was a high-fat meal, it could be inferred that the dietary fatty acids cause the CCK release and that the fatty acids are formed during a longer time period compared to control. Thus thylakoids slow down digestion of dietary fat in man in a way that promotes the release of CCK during a longer period of time. A further proof of delayed fat digestion was a late appearance of fatty acids in the blood compared to control [31]. During these studies leptin was significantly elevated compared to control at time point 6 h [31]. Since delipidated thylakoids inhibit lipase as efficient as normal thylakoids [18] it was of interest to study their potential appetite suppressive effect. The release of CCK was similar as for normal thylakoids; however, there was no release of leptin [31]. Delipidated thylakoids, lacking pigments and membrane lipids, thus are not as efficient to regulate appetite as untreated thylakoids.

Since thylakoids induce satiety in fat-rich meals, an obvious question was if thylakoids also act as appetite suppressor in carbohydrate-rich meals. Overweight women were served a carbohydrate-rich breakfast with and without thylakoids [33]. The meal consisted of muesli, white bread, butter, cheese, ham, black current jam, yoghurt, orange juice, and banana together with coffee and was followed during 4 h. The meal consisted of 71 E% carbohydrate, 11 E% fat and 18 E% protein by energy. The thylakoids were mixed into the jam and eaten with yoghurt and muesli. The study had a crossover design. The overweight women experienced a suppressed hunger and motivation to eat after consumption of the thylakoid-enriched breakfast compared to control breakfast [33]. The appetite suppression became apparent after 2 h from the start of the breakfast. A significant elevation of CCK levels were registered from 3 h and onwards compared to control. The study concluded that thylakoids were able to give a late satiety and suppressed hunger motivation, even with a high-carbohydrate meal [33]. It is important to note that the hunger was suppressed and satiety promoted by thylakoids as early as 2 h after the start of meal, measured in a standard carbohydrate-based breakfast [42].

The explanation for a postprandial satiety even with a high-carbohydrate, low-fat meal suggests that other mechanisms for the appetite suppressive effect of thylakoids unrelated to a reduced rate of fat digestion may be relevant. One such mechanism could be the effect of thylakoids on glucose homeostasis and insulin secretion. The thylakoid-enriched breakfast demonstrated a biphasic response of glucose levels in the blood, whereas the control breakfast demonstrated a single rise of glucose, followed by a hypoglycaemia postprandially [33]. The hypoglycaemia in the control breakfast correlated with an increased hunger. Hypoglycemia is known to stimulate hunger, whereas a rise in blood glucose causes satiety [43].

**Fig. 4:**
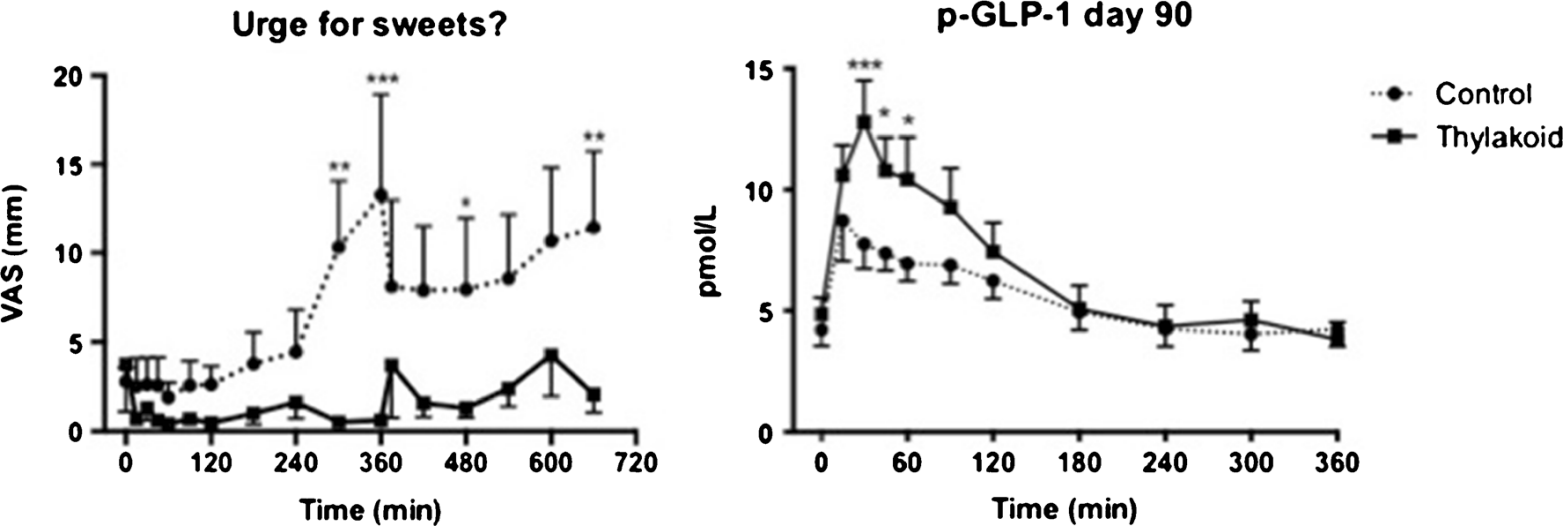
Suppressed urge for sweet and release of GLP-1 (glucagon-like peptide 1) with and without the consumption of thylakoids. Overweight women were served a breakfast at time point zero with or without 5 g of thylakoids and a lunch at time point 360 min. Urge for sweet was measured through VAS as well as GLP-1 through blood sampling. The subjects receiving thylakoids had a significantly suppressed urge for sweet and increased release of GLP-1 even after 90 days of body weight loss compared to control [41]

Another explanation for the increased satiety by thylakoids may be the release of CCK and GLP-1. Release of CCK has been postulated to occur only after fat- and protein-rich meals. Even a carbohydrate-rich meal was found to raise CCK levels [44]. Release of GLP-1 was significantly stimulated by thylakoids during consumption of a breakfast in overweight women (Fig. 4) [41]. GLP-1 is a satiety hormone, produced by intestinal cells in response to carbohydrate and fat. In particular fatty acids are potent releasers of GLP-1. GLP-1 is a hormone that is being developed as an anti-obesity drug. In our hands thylakoids caused the endogenous release of GLP-1, which is an easier and more physiological way to enhance GLP-1 levels to achieve satiety and energy balance.

A suppression of the hunger signal ghrelin by thylakoids was observed after the high-dose thylakoid in a single meal consisting of 60 % fat and where the thylakoids were added in a pesto sauce [31]. Ghrelin was like-wise suppressed in pig receiving pure carbohydrate by thylakoids compared to control [45]. The effect of thylakoids on appetite regulation and appetite regulation hormones occurs thus with both carbohydrate- and fat- rich meals.

### Suppression of Hedonic Hunger

Hedonic hunger is characterized by 1) thinking of food, although you have just eaten, 2) an uncontrolled urge to eat highly palatable food, containing sugar, salt or fat and 3) overeating. Overweight subjects have an increased liking of palatable food, in particular sugar [46] and fat [47]. It is hence of great importance to find strategies that may promote control of hedonic hunger.

We found that thylakoids indeed suppressed hedonic hunger [41, 42, 48]. Overweight women were served a single breakfast with or without a prior shot with thylakoids. The liking for salt, sweet, fat and sweet/fat was estimated using a VAS scale combined with pictures (chips, goodies, cheese on bread and chocolate). A significant reduction in the liking of fat and sweet was demonstrated after a single shot of thylakoids, the same day [41, 42, 48]. The liking decreased further after 12 weeks of daily consumption of 5 g thylakoids [41] (Fig. 4). It is concluded that thylakoids have the ability to suppress urge for sweet and fat. This appears to last the whole day following consumption of a thylakoid-enriched juice in the morning [42]. This is particularly important since cravings for sweet usually start in the afternoon. Thylakoids when consumed in this way thus provides a powerful strategy to suppress the urge for palatable food. In the long-term studies GLP-1 was significantly increased by thylakoids, both at the first day, but even more the last day of thylakoid treatment [41] (Fig. 4). Since GLP-1 is a strong suppressor of urge for sweet food, the increase in GLP-1 may well explain the observed decreased sweet urge. The mechanism may also involve the satiety hormone CCK. Another explanation may be the stabilization of serum glucose by thylakoids to prevent postprandial hypoglycaemia, which otherwise triggers craving for rewarding food.

### Loss of Body Weight and Body Fat

Various experiments demonstrate that thylakoids promote weight loss in rat, mouse and in man. In rat a loss of 17,5 % body weight (*p* < 0,05) was noted after 13 days of treatment with thylakoids added to a high-fat diet [18]. The loss of body weight was associated with a reduction of serum triacylglycerol by 40 % (*p* < 0,05).

In mice body weight loss with thylakoids was 17 % (*p* < 0,001) compared to control mice after 100 days of treatment [32]. The weight loss occurred with a reduced food intake (*p* < 0,001) and an increased release of the satiety hormone CCK, being increased by 52 % (*p* < 0,05). The loss in body weight was evident from day 28. The time delay suggests that genes are gradually upregulated that promote satiety signalling. Another explanation is a changed bacteria flora as evidenced after administration of thylakoids to rat [40]. Whether an increased thermogenesis is induced by thylakoids is not known.

The thylakoid-diet given to mice also reduced body fat mass by approximately 33 % (*p* < 0,001) and serum leptin by 44 % (*p* < 0,01) [32]. The loss in body weight was thus effectively a loss of fat mass, evidenced both by tissue measurements using a DEXA-scanner and by leptin measurements, leptin being representative for the amount of fat mass [49]. The loss of body fat suggests that there is an increased fatty acid oxidation by thylakoids.

In these studies serum triacylglycerol was at the same time reduced by 25 % (*p* < 0,05) and serum glucose by 17 % (*p* < 0,05), supporting the coupling between body weight loss, fat mass loss and reduction of serum lipid/glucose values [32]. Somewhat surprising the levels of serum free fatty acids were also reduced after thylakoid treatment by 16 % (*p* < 0,05). It is remarkable that the levels of free fatty acids were reduced by the thylakoid diet compared to control. A loss of body weight by restricted diet is often associated with an increase in the levels of free fatty acids. Free fatty acids have a potential to cause inflammation in pancreas, liver and the brain [26]. The reduction of free fatty acids by thylakoids during weight loss may thus be an important health-promoting property.

In human, thylakoids were shown to promote body weight loss together with reduction of blood lipids, significantly different from control (*p* < 0,02) [41]. Thirty-eight women (40–65 years of age) with a BMI of 25–33 were randomly assigned to consume a glass of blueberry juice prior to breakfast with thylakoids (5 g/day) or without for 12 weeks. They were recommended to eat three meals a day and exert 30 min of physical activity daily. At the end of the study, both groups had lost body weight, but significantly more in the thylakoid-treated group, −5,0 kg in the thylakoid group *versus* −3,5 kg in the control group (*p* < 0,01). Coupled with weight loss the most significant changes induced by thylakoids compared to control was a reduction in LDL-cholesterol (*p* < 0,05) levels. This decrease occurred already after three weeks, thus prior to the body weight loss. This suggests that thylakoids lower blood lipids by other mechanisms than a weight-loss consequence. The effects suggest that thylakoids may be helpful for treatment of obesity and metabolic syndrome.

### Promotion of Glucose Homeostasis

Hyperglycaemia is a major symptom in type-2-diabetes, the most prevalent disease affecting individuals with a westernized life-style. Prevention of hyperglycaemia is achieved through the consumption of low-glycaemic food in contrast to high-glycaemic food, the uses of inhibitors of carbohydrate digestion, and the use of medical drugs that stimulate the uptake of glucose from the blood into peripheral cells. When using low-glycaemic food fibres present therein bind starch, in this way slowing down the digestion of starch into glucose. Inhibitors of starch digestion are present naturally in the plant kingdom. One such inhibitor is proantocyanidin in cinnamon, demonstrated to inhibit carbohydrate hydrolysis *in vitro* [50]. This is an important effect to explain the blood glucose lowering effect of cinnamon. Whether the antioxidants present in thylakoids have any inhibiting capacity on amylase activity, is not known. Regarding blood glucose it is important also to control the absorption process. The absorption of glucose occurs through the apical brush-border membrane of the intestinal epithelial cell, mainly through the action of the sodium-glucose linked transporter I. In rat intestine, glucose uptake was significantly reduced by the presence of thylakoids *in vitro* [8]. The explanation for the reduced uptake of glucose may be the localization of thylakoids as large complex structures onto the mucosa [8]. There may also be a binding of thylakoids to the starch and/or amylase. *In vivo*, a 10-day treatment with thylakoids in rat resulted in reduced glucose levels after an oral glucose tolerance test [40]. This suggests an improved glucose homeostasis induced by thylakoids. Thylakoids also decreased blood glucose levels during long-term treatment in rat [40], mouse [32] and pig [51]. Theses changes were related to the negative energy balance following weight loss. Weight loss treatment in general leads to improvement of glucose homeostasis, the development of obesity often being associated with type 2 diabetes mellitus [1]. According to the first law of thermodynamics, reduced food consumption spontaneously creates a negative energy balance, with an improvement of the metabolic profile of the overweight person. Thus, weight loss of 5–10 kg is sufficient to decrease plasma levels of glucose, insulin, and blood lipids/lipoproteins [51]. Thylakoids may thus be helpful during the treatment of obesity and type-2-diabetes to reduce blood glucose levels.

### Reduction of Blood Triglycerides and Cholesterol

A consistent finding with long-term treatment with thylakoids is a reduction of blood lipids, both triacylglycerol and cholesterol. In rat, triacylglycerol levels were reduced by 40 % following a 13-day treatment with thylakoid-enriched food [18] and in mice reduced by 24 % following a 100–day treatment [32]. The reduction in lipid levels occurred simultaneously with a loss of body weight. A 15 % reduction of triacylglycerol levels is a common goal for treatment of hypertriglyceridemia [52]. Thylakoids thus have a potential to be used as an alternative for lipid-reducing drugs. The mechanism for the reduced levels of blood lipids is not known. Perhaps a stimulated fatty acid oxidation occurs through the presence of pigments in thylakoids [16]. Cholesterol-lowering effect may be related to bile salt binding, thereby inhibiting the intestinal absorption [53]. Another mechanism may be the prebiotic effect of thylakoids [40]. *Lactobacillus reuteri*, a strain increased by thylakoids in rat, has been demonstrated to decrease serum cholesterol levels [54].

### Prebiotic Effect of Thylakoids

Dysregulation of energy metabolism as occurs in obesity and diabetes may be related to gut microbiota [55]. Gut microbiota process components of the diet that have not been digested in the small intestine. They hence take part in nutrient acquisition and energy regulation. By producing metabolically active components they also affect glucose homeostasis as well as lipid metabolism. Some bacteria even act as anti-obesity factors, like lactobacilli and bifidobacteria [55, 56]. Thylakoids when administered to rat for 10 days modulated the gut microbiota [40]. *Lactobacillus reuteri* was significantly increased in the distal ileum compared to control rats, whereas bifidobacteria were not changed [40]. *Lactobacillus reuteri* was shown to prevent obesity in apo-E-deficient mice given a high-fat diet [57]. Using the same strain of mice we found that thylakoids prevented weight gain during high-fat diet without affecting the cholesterol levels [32], suggesting that the anti-obesity effect of thylakoids may be linked to a change in microbiota. Also in human thylakoids affect the microbiota, as measured during a 10-week study with and without thylakoids were changed (C. Montelius, unpublished results). The change in microbiota may contribute to the anti-obesity effect of thylakoids in human and the improvement of lipid and glucose homeostasis.

### Suppression of Inflammation

Suppression of inflammation is important to prevent aging diseases, such as atherosclerosis. Berries with their antioxidants have been found extremely useful to provide a suppression of inflammation [58]. Since thylakoids contain antioxidants we were interested whether these had any effect on inflammation. TNF-alpha was suppressed after a single meal (*p* < 0,01) [33]. Whether such a suppression is observed after long-time is not known, but is an important issue for long-term health benefit.

## Summary

**Fig. 5:**
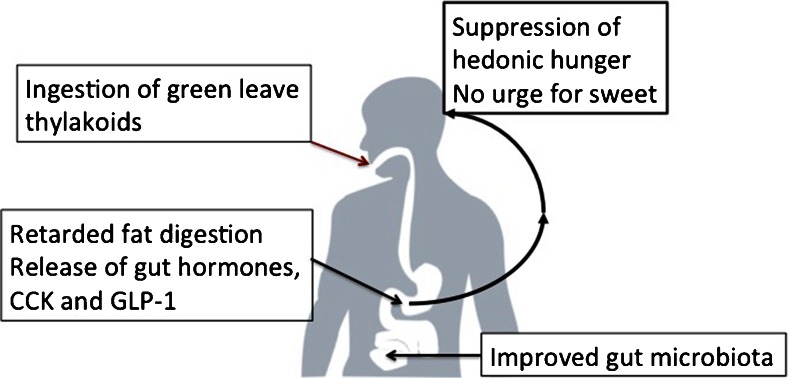
The mechanism of action of thylakoids in appetite regulation. When thylakoids are consumed they will retard fat digestion, and release the gut hormones cholecystokinin (CCK) and glucagon-like peptide-1 (GLP-1). These hormones promote satiety and suppress hunger, in particular hedonic hunger *i.e*., urge for sweet and fat. The thylakoids also change the bacteria in the intestine in a prebiotic way, increasing the strains *Lactobacillus reuteri*. With these effects thylakoids are important for prevention of obesity and diabetes

In conclusion, thylakoids when added to food are powerful promoters of satiety and suppressors of hunger, in particular hedonic hunger. The mechanism is a late satiety, by the use of the whole intestine for digestion of food. In this way satiety hormones are released from the distal intestine, the ileal break, providing information to the brain that satiety has been achieved and a reduced urge for snacking. Since snacking is an important contributor to the global epidemic of obesity today, the consumption of thylakoids may aid in the prevention of this epidemic. The effects of thylakoids are summarized in Fig. 5.

## Abbreviations

CCK: Cholecystokinin
DEXA: Dual-energy X-ray absorptiometry
GLP-1: Glukagon-like peptide-1
PPARs: Peroxisome proliferator-activated receptors
TNF-alfa: Tumour necrotic factor-alfa
VAS: Visual analogous scale
